# Upper Limb Kinematics in Stroke and Healthy Controls Using Target-to-Target Task in Virtual Reality

**DOI:** 10.3389/fneur.2018.00300

**Published:** 2018-05-09

**Authors:** Netha Hussain, Margit Alt Murphy, Katharina S. Sunnerhagen

**Affiliations:** Institute of Neuroscience and Physiology, Sahlgrenska Academy, University of Gothenburg, Gothenburg, Sweden

**Keywords:** stroke, rehabilitation, upper extremity, movement, kinematics, outcome assessment, virtual reality, discriminative validity

## Abstract

**Background:**

Kinematic analysis using virtual reality (VR) environment provides quantitative assessment of upper limb movements. This technique has rarely been used in evaluating motor function in stroke despite its availability in stroke rehabilitation.

**Objective:**

To determine the discriminative validity of VR-based kinematics during target-to-target pointing task in individuals with mild or moderate arm impairment following stroke and in healthy controls.

**Methods:**

Sixty-seven participants with moderate (32–57 points) or mild (58–65 points) stroke impairment as assessed with Fugl-Meyer Assessment for Upper Extremity were included from the Stroke Arm Longitudinal study at the University of Gothenburg—SALGOT cohort of non-selected individuals within the first year of stroke. The stroke groups and 43 healthy controls performed the target-to-target pointing task, where 32 circular targets appear one after the other and disappear when pointed at by the haptic handheld stylus in a three-dimensional VR environment. The kinematic parameters captured by the stylus included movement time, velocities, and smoothness of movement.

**Results:**

The movement time, mean velocity, and peak velocity were discriminative between groups with moderate and mild stroke impairment and healthy controls. The movement time was longer and mean and peak velocity were lower for individuals with stroke. The number of velocity peaks, representing smoothness, was also discriminative and significantly higher in both stroke groups (mild, moderate) compared to controls. Movement trajectories in stroke more frequently showed clustering (spider’s web) close to the target indicating deficits in movement precision.

**Conclusion:**

The target-to-target pointing task can provide valuable and specific information about sensorimotor impairment of the upper limb following stroke that might not be captured using traditional clinical scale.

**Trial registration details:**

The trial was registered with register number NCT01115348 at clinicaltrials.gov, on May 4, 2010. URL: https://clinicaltrials.gov/ct2/show/NCT01115348.

## Introduction

In stroke, the prevalence of upper limb impairment is approximately 50–80% in the acute phase (1–3) and 40–50% in the chronic phase (2, 4). The frequently observed upper limb impairments after stroke are paresis, abnormal muscle tone, decreased somatosensation, and coordination. As a consequence of these impairments, individuals with stroke may experience reduced ability to perform everyday activities such as opening a door, handling a key, or working with a computer. Therefore, assessment of upper limb motor function is critical for determining the prognosis and evaluating the treatment effects following stroke (5, 6).

The assessment of motor functions in stroke is usually performed using standardized clinical scales. Some of the most frequently used clinical instruments for assessing upper extremity impairment and activity capacity in stroke are Fugl-Meyer Assessment of Upper Extremity (FMA-UE) and Action Research Arm Test (ARAT) (7–9). These scales are reliable (10–12) and responsive to change (13, 14) for measuring gross changes in motor function. They have also been recommended as core measures to be included in every stroke recovery trial (6). However, observer-based ordinal instruments like FMA-UE and ARAT lack the sensitivity to assess subtle, yet, potentially important changes in movement performance (15). These clinical scales tend to have ceiling effect since they rely on scoring criteria rather than a continuous measurement construct (16).

Kinematic assessment is one solution for the need for a more objective, accurate, and sensitive measurement method (6). Kinematic assessment is being increasingly used in upper limb evaluation after stroke, out of which motion capture systems (17), robotic devices, and virtual reality (VR) systems with haptic devices (18) have become popular in the last decade. Kinematic assessment has revealed that the arm movements in subjects with stroke are slower, less accurate, less smooth, and more segmented than healthy subjects (19–26).

Kinematic assessment involving the use of VR with haptic device has shown to be a promising tool for upper limb stroke rehabilitation (27, 28). Despite the availability of the VR system for stroke rehabilitation, it has been rarely used in assessment of upper limb movements after stroke. Individuals with stroke use similar strategies for reaching objects in both real and virtual environments (29). Previous studies using the target-to-target pointing task have shown that the movement time, velocity, and trajectory straightness were improved after a 5-week computer gaming practice in individuals with stroke (30). Movement time, mean velocity, and trajectory straightness were also stable in a test–retest study in healthy subjects (31). A clear advantage with VR systems as a measurement tool is its standardized instructions, adaptation of tasks according to patients’ functioning level, and availability of quick feedback (32). The VR assessment and training are often described as enjoyable and challenging by the users (33, 34).

The target-to-target pointing task is similar to routinely used tasks in everyday life, such as interacting with touch screens, using electrical switches, and pushing buttons on various devices. The choice of a regularly performed, purposeful task for this study increases its ecological validity. With VR technologies becoming more available, it opens up an opportunity to use the VR interface to acquire accurate and detailed kinematic data of upper limb movements after stroke (35). The novelty of this study is in evaluating a compact and easy-to-use haptic device coupled with VR in 3D space in order to measure movement performance during a common upper limb task.

The aim of this study was to identify the end-point kinematic variables obtained during the VR-based target-to-target pointing task that discriminate among individuals with mild and moderate upper limb impairment after stroke and healthy controls.

## Materials and Methods

### Subjects and Design

Data for this study were extracted from Stroke Arm Longitudinal Study at Gothenburg University (SALGOT; https://ClinicalTrials.gov, identifier: NCT01115348) (36), a longitudinal, prospective, observational study. The SALGOT cohort comprised of a non-selected population of 122 adults living in Gothenburg urban area, with first-ever clinical stroke and stroke-related impaired upper limb function, admitted to the stroke unit within 3 days of stroke onset. The diagnosis of stroke was based on World Health Organization (WHO) collaborative study criteria (ischemic infarct and hemorrhagic) (37). Each individual was assessed using a battery of tests eight times during the first year of stroke, at 3, 10 days, 3, 4, and 6 weeks, and 3, 6, and 12 months after the onset of stroke (36). The exclusion criteria of SALGOT were: upper limb condition prior to stroke that limits the functional use of the arm, severe multi-impairment, or other diseases and conditions of arm before stroke that will affect arm function, life expectancy less than 12 months due to severity of stroke injury or other illnesses and not Swedish speaking prior to the stroke incident.

**Figure 1:**
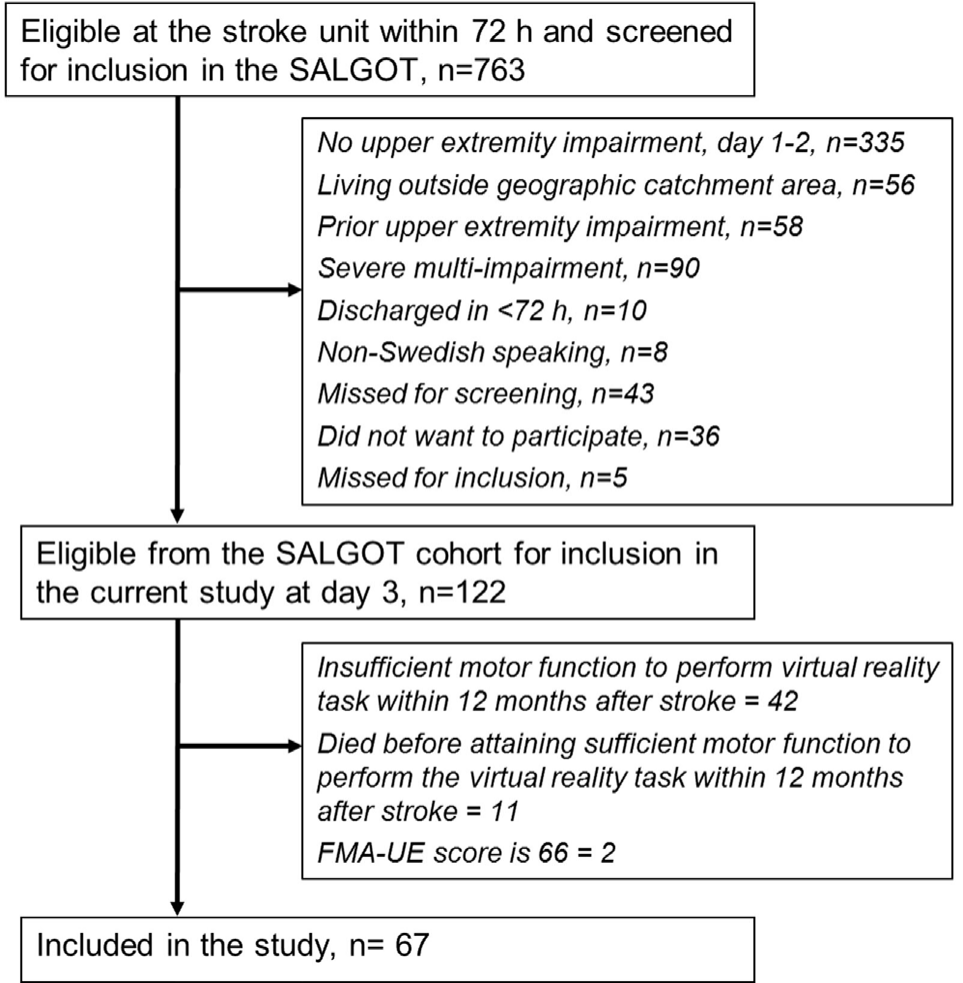
Flowchart of the inclusion process. Abbreviations: SALGOT, Stroke Arm Longitudinal Study at Gothenburg University; FMA-UE, Fugl-Meyer Assessment for upper extremity.

The participants from SALGOT cohort were included in the present study if they had sufficient motor function to perform the target-to-target pointing task and had not reached a full score of 66 points on the FMA-UE scale at least at one testing occasion during the first year poststroke. In cases where a participant had performed the pointing task during more than one testing occasion, the kinematic data from the first occasion only were included in the study. In this way, the learning effect that might occur after testing multiple times for the SALGOT trial was eliminated. In total, 67 individuals (28 women) with stroke met these inclusion criteria and were included in the present study. The flowchart of the inclusion process is shown in Figure 1.

The control group included 43 individuals with commensurable age (mean and SD = 64 ± 14 years) and gender distribution (20 women). They perceived themselves as healthy and reported to have no neurological or musculoskeletal disorders affecting upper limb movements. They were excluded from the study if they were unable to follow instructions in Swedish/English or had uncorrected visual acuity or hearing loss that influenced the test performance. Kinematic assessment was done only once in individuals from the control group.

The SALGOT study protocol that included the present study was approved by the Regional Ethical Review Board in Gothenburg, Sweden (No. 318-04, 225-08). All participants gave informed, written consent prior to their participation in the study. Participants were given full information about the assessment procedure and were free to discontinue their participation without having to provide an explanation. They were allowed to rest between assessments whenever needed. They were free to discuss the issues that arose during the testing with the test leader.

The power of SALGOT trial was set at 0.8 with a significance level of 0.05 (two-sided test), such that a clinically significant change of 6 points (10%) can be detected on ARAT. The required sample size was calculated to be 120, assuming a 30% dropout (36).

### Equipment

**Figure 2:**
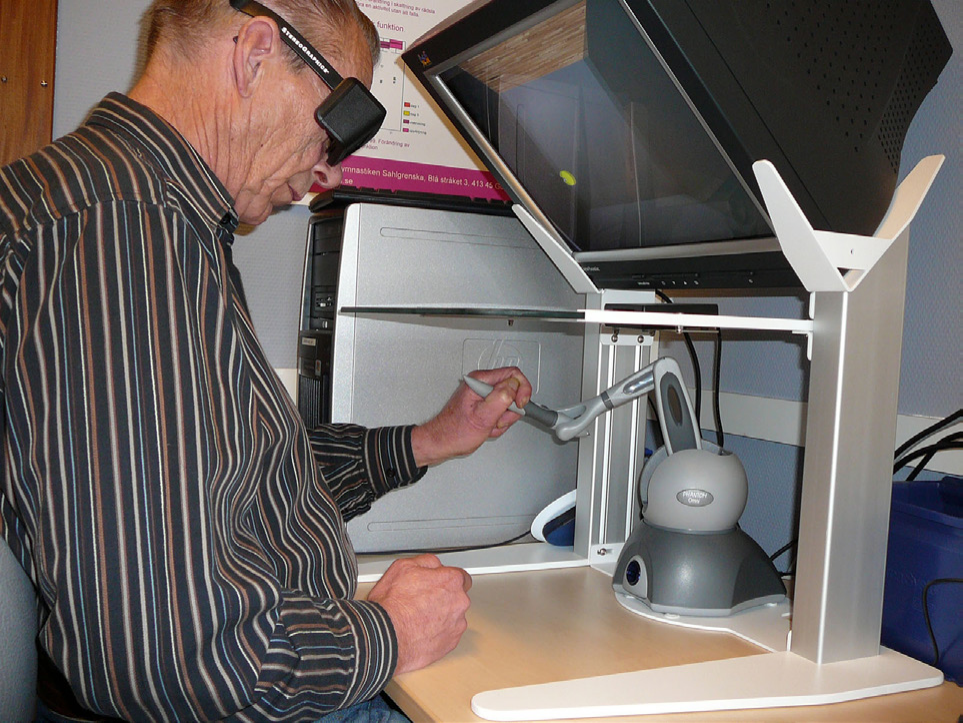
A participant wearing 3D glasses and performing the target-to-target task using the haptic device.

The VR environment included a semi-immersive workbench in which 3D display of virtual space is observed by the user on a mirror after wearing stereographic shuttered glasses (Figure 2). An infrared receiver located on the center of the glasses worked with the infrared transmitter on the workbench and synchronized the right and left image sequence on display, giving the participant an illusion of seeing 3D objects.

The PHANTOM^®^ Omni™ haptic device was used to capture kinematic data. It included a stylus that has 6 degrees of freedom (*x, y*, and *z* coordinates, pitch, roll, and yaw). The haptic stylus could be moved freely in the virtual workspace (160 mm × 120 mm × 120 mm) and its nominal position resolution was 0.055 mm. The device was autocalibrated when the stylus was placed in its resting position. The user received force feedback when the stylus was brought close to the target. A pressure of at least 0.5 N had to be given by the participant close to the target in order to manipulate the virtual target. This setup created an illusion of touching the virtual target. Additionally, auditory and visual (color change and disappearance) feedback was also given when the virtual target was pointed at by the participant.

### Set-Up and Procedure

The kinematic data were gathered from the haptic device during the target-to-target pointing task. The participant was instructed to sit comfortably on a chair without armrest and wear 3D glasses. He/she was asked to hold the stylus with one hand using pen grip, although alterations in grip was allowed and was noted for the record. Participant was then asked to reach and point at a green colored, disk-shaped target (~3.0° viewing angle) that appeared and disappeared when pointed at by the tip of the stylus. All targets were displayed in the participant’s virtual workspace and they did not have to lean forward to reach them. The task consisted of 32 targets and each new target appeared after the previous one was pointed at and made to disappear. The task ended when the last of 32 targets disappeared.

Though the locations of the targets appeared to be random for the participant, they were actually predetermined by the software for uniformity and ease of evaluation. The targets were distributed non-uniformly in the 3D space, with the shortest movement segment measuring 76 mm and the longest segment measuring 180 mm. The participants needed to reach each target with the tip of the stylus, one at a time, in order to complete the task. There was no time limitation for the task and several attempts within each segment were allowed. The participant was asked to finish the task as fast and as accurately as possible, first with the unaffected arm followed by the affected arm. Healthy controls performed the task in random order of the arms. The participant was permitted to perform one or two trials before the assessment to acquaint themselves with the VR setup. The task had no constraints with respect to velocity of movement or total time taken for its completion.

### Kinematic Variables

Each recording obtained from the movement of the stylus was time stamped and the kinematic variables were calculated using custom-made Curictus™ and MATLAB^®^ software. The sampling frequency was 50 Hz. The data were filtered with a 6-Hz low-pass second order Butterworth filter in both forward and reverse directions. The entity between two adjacent targets was defined as a movement segment. A movement segment begins when a new target appears and ends when it is pointed with the stylus, and made to disappear. The first movement segment was between the first and second targets. Thus, the entire target-to-target task included 31 such segments. Five kinematic variables were calculated: movement time, mean velocity, peak velocity, time to peak velocity, and number of velocity peaks. Each kinematic variable was calculated as means of all 31 movement segments for the entire task.

Movement time was defined as the time taken to complete one movement segment. The maximum absolute velocity recorded during each movement segment was taken as peak velocity. Mean velocity was calculated separately for each movement segment. The time to peak velocity was expressed in percentage of movement time and reflects the movement strategy. This measure distinguishes between the relative time spent during the first visually triggered outward movement until the peak velocity is reached, and the second half of the movement toward the target that requires more precision in order to touch the target.

The smoothness of movement was measured by counting the number of velocity peaks in a movement segment. In order to define a peak, the velocity profile was first searched for local minima and maxima. When the difference between a minimum and the next maximum exceeded the cut-off limit of 20 mm/s, it was counted as a velocity peak. Additionally, the time between two subsequent peaks had to be at least 150 ms.

The choice of these variables, and criteria for computing peaks were based on previous studies (26, 31) and the observations made on graphical representation of kinematic data. The movement time, measures of velocity, and smoothness have previously been proposed in literature as key kinematic variables for end-point performance in individuals with stroke (17, 27).

### Clinical Assessments

Fugl-Meyer assessment of upper extremity is a stroke-specific scale used for assessing the sensorimotor function of upper limb. It has been widely used to evaluate the recovery of upper limb in poststroke individuals. This scale assesses the ability to perform isolated movements within and outside of synergies (7). FMA-UE is valid (16) and reliable (10, 11) for upper limb assessment in stroke. A maximum score of 66 in FMA-UE indicates normal arm function. The non-motor domains of FMA-UE (sensation, passive joint motion, and pain) were also recorded for background data. Participants with stroke were dichotomized into moderate (FMA-UE score: 32–57 points) and mild (FMA-UE score: 58–65 points) stroke impairment subgroups (15, 26, 38–40). NIHSS scoring (41) was done to determine the type and severity of stroke at the time of hospital admission. The background data of the participants with stroke are summarized in Table 1.

**Table 1:** Demographic data and clinical characteristics of individuals with stroke.

Characteristics Mean ± SD, *n*(%) or median (IQR)	Moderate stroke (*n* = 33)	Mild stroke (*n* = 34)	All stroke (*n* = 67)
Age	66.8 ± 12.0	64.7 ± 14.7	65.7 ± 13.4
Female	15 (46%)	13 (38%)	28 (42%)
Time since stroke median (in months)	0.3 (0.9)	0.3 (0.2)	0.3 (0.9)
day 3/day 10	12/5	19/11	31/16
week 3/week 4/week 6	2/6/3	1/1/0	3/7/3
month 3/month 6/month 12	3/1/1	2/0/0	5/1/1
Ischemic stroke	22 (67%)	32 (94%)	54 (81%)
Hemorrhagic stroke	11 (33%)	2 (6%)	13 (19%)
NIHSS motor arm score, 0/1/2/3/4, *n* = 65	6/11/1/4/11	5/18/2/4/3	11/29/3/8/14
Right hand dominant	33 (100%)	31 (95%)	64 (97%)
Right hemiparesis	14 (42%)	15 (43%)	29 (43%)
Left hemiparesis	19 (58%)	19 (57%)	38 (57%)
Fugl-Meyer assessment—upper extremity (0–66)	51 (14)	61.5 (5)	58 (10)
Decreased sensation (≤11 points, FMA)	12 (37%)	6 (18%)	18 (27%)
Impaired passive joint motion (≤23 points, FMA)	13 (41%)	3 (9%)	16 (24%)
Pain during passive movements (≤23 points, FMA)	10 (31%)	5 (15%)	15 (22%)
Spasticity of elbow or wrist joint (≥1 point, MAS)	4 (12%)	1 (3%)	5 (8%)

*IQR, interquartile range; FMA, Fugl-Meyer Assessment of sensorimotor function*.

### Statistical Data Analysis

Statistical analyses were performed using IBM Statistical Package for Social Sciences™ (SPSS) version 24 for Windows. The level of significance was set to *p* < 0.05 (two-tailed). The normality of distribution of all variables were tested using Shapiro–Wilk’s test.

Since a majority of variables were non-normally distributed, non-parametric statistics were used. Wilcoxon’s signed rank test was performed for comparing the dominant and non-dominant arm of healthy controls. Age and height of the participants were tested for significant differences using the Mann–Whitney *U* test. Determination of significant differences between all individuals with stroke and healthy controls was also done by Mann–Whitney *U* test. To determine whether there were significant differences between the three groups (moderate stroke impairment, mild stroke impairment, and healthy controls), Kruskal–Wallis one-way analysis of variance was used. Whenever significant differences were obtained in Kruskal–Wallis test, Mann–Whitney *U* test with Bonferroni correction was used for *post hoc* testing. Thus, the *p*-value required for significance was adjusted to 0.017 in such cases.

The strength of differences between groups was determined using effect size estimates. Point biserial correlation (*r*_pb_) was used to calculate effect size since non-parametric tests were used to test the hypotheses (42). Cohen’s guidelines were followed while interpreting the effect sizes, where 0.1, 0.3, and 0.5 indicate small, medium, and large effect sizes, respectively (42). For those variables that showed significant differences between individuals with stroke and healthy controls, sensitivity and specificity were also calculated. Sensitivity refers to the proportion of individuals with stroke diagnosis who are correctly identified as having stroke by the cut-off values for the kinematic variables. Specificity refers to the proportion of healthy controls who are correctly identified as controls by the cut-off values for kinematic variables (43). In this study, the cut-off values for determining sensitivity and specificity were determined as one SD of the respective mean value of kinematic variable of the healthy controls (26).

## Results

There were no significant differences in age (65.7 ± 13.4 years for stroke group and 64.9 ± 14.1 for control group, *p* = 0.71) and height (*p* = 0.85) between individuals with stroke and healthy controls. Similarly, the age and height differences were not significant between moderate stroke and mild stroke groups (*p* = 0.36 and 0.52, respectively). There were no significant differences between dominant and non-dominant upper limbs of healthy controls in terms of all measured kinematic variables. However, the non-dominant arm of healthy controls was chosen for calculating the group statistics to avoid putting those in the stroke group who have hemiparesis in the non-dominant arm to a comparative disadvantage.

### Movement Time

The stroke group had significantly longer movement times when compared to healthy controls (*p* < 0.001, *r*_pb_ = 0.72). For a cut off of 1.56 s (mean + one SD of healthy controls), the sensitivity for discriminating between stroke group and healthy controls was 82.1% and specificity was 86.1%. The differences in movement times were also significant between mild and moderate stroke groups (*p* = 0.003, *r*_pb_ = 0.36), mild stroke group and healthy controls (*p* < 0.001, *r*_pb_ = 0.65), as well as moderate stroke group and healthy controls (*p* < 0.001, *r*_pb_ = 0.81). The mean values and SD for the kinematic variables are shown in Table 2.

**Table 2:** Mean and SD of kinematic variables for all participants during the target-to-target task.

	Moderate stroke (*n* = 33)	Mild stroke (*n* = 34)	All stroke (*n* = 67)	Healthy controls (*n* = 43)
Movement time (s)	3.21 (2.24)^a,c^	2.33 (1.52)^b^	2.80 (1.97)^d^	1.31 (0.25)
Mean velocity (mm/s)	119.2 (43.80)^a,c^	165.7 (62.92)^b^	144.8 (59.74)^d^	209.2 (48.86)
Peak velocity (mm/s)	330.7 (104.3)^a,c^	417.8 (147.69)	374.8 (134.6)^d^	440.7 (91.81)
Time to peak velocity (%)	29.09 (7.15)	33.52 (11.63)	31.34 (9.87)	33.29 (10.51)
Number of velocity peaks	5.24 (2.90)^c^	4.29 (2.33)^b^	4.76 (2.65)^d^	2.80 (0.53)

*^a^p < 0.01 between moderate and mild stroke*.

*^b^p < 0.01 between mild stroke and non-dominant arm in healthy controls*.

*^c^p < 0.001 between moderate stroke and non-dominant arm in healthy controls*.

*^d^p < 0.001 between all stroke and healthy controls*.

### Movement Velocity

The stroke group had significantly lower mean velocity than healthy controls (*p* < 0.001, *r*_pb_ = 0.53). Sensitivity and specificity of mean velocity were 70.2 and 83.7%, respectively. The mean velocity was significantly different across mild and moderate stroke groups (*p* = 0.002, *r*_pb_ = 0.39), mild stroke group and healthy controls (*p* = 0.001, *r*_pb_ = 0.38), and moderate stroke group and healthy controls (*p* < 0.001, *r*_pb_ = 0.72).

**Figure 3:**
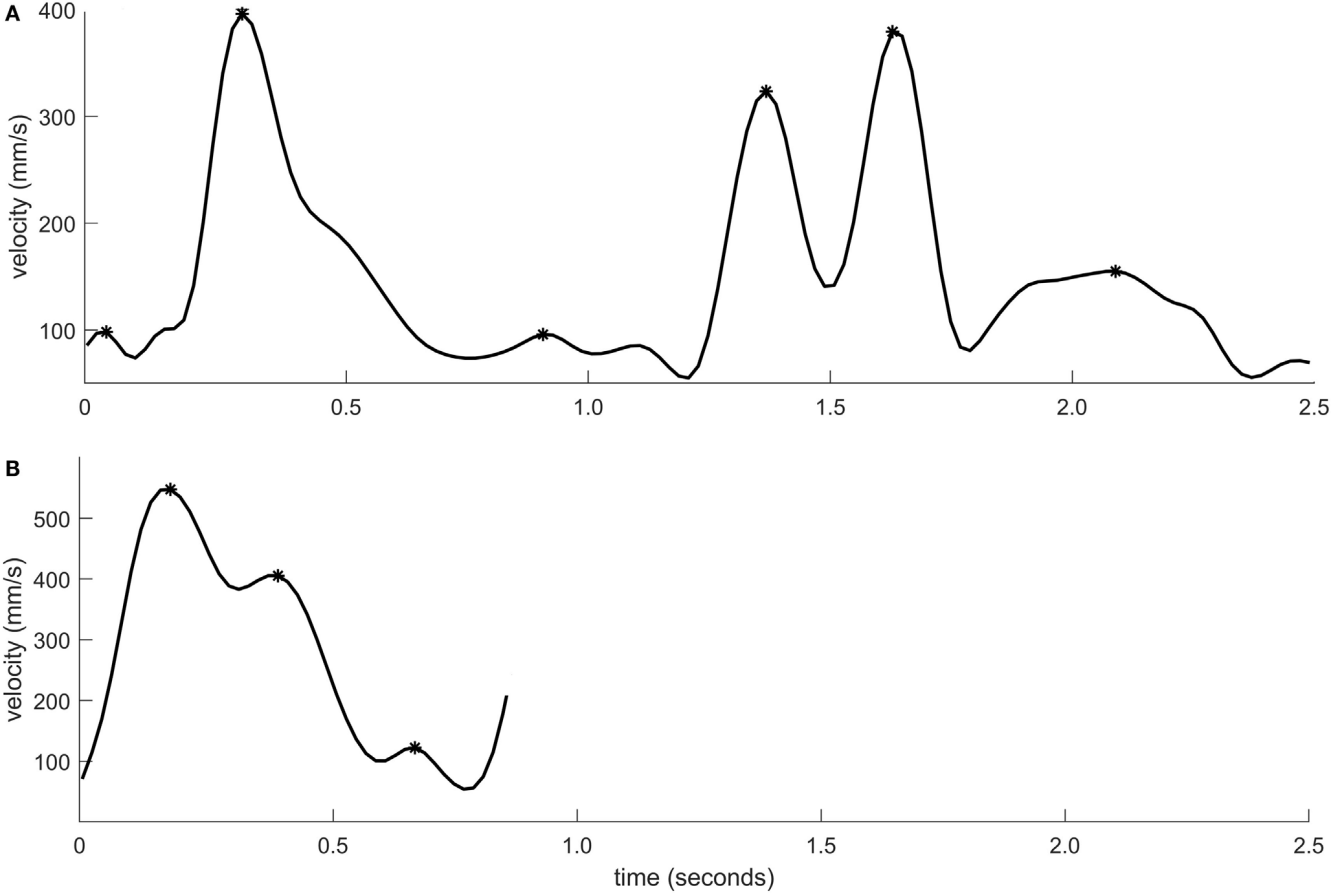
Velocity–time profile of a movement segment shown for one individual with moderate stroke impairment **(A)** and one healthy control **(B)**. The velocity peaks are marked using asterisk symbols.

Peak velocity was significantly lower for individuals with stroke when compared to healthy controls (*p* < 0.001, *r*_pb_ = 0.38). Peak velocity was also significantly lower in moderate stroke group in comparison with mild stroke group (*p* = 0.004, *r*_pb_ = 0.35). The sensitivity was 46.3% and specificity was 93.0%. Figure 3 shows the velocity–time curve of one representative participant each from stroke group and healthy controls.

### Movement Strategy

**Figure 4:**
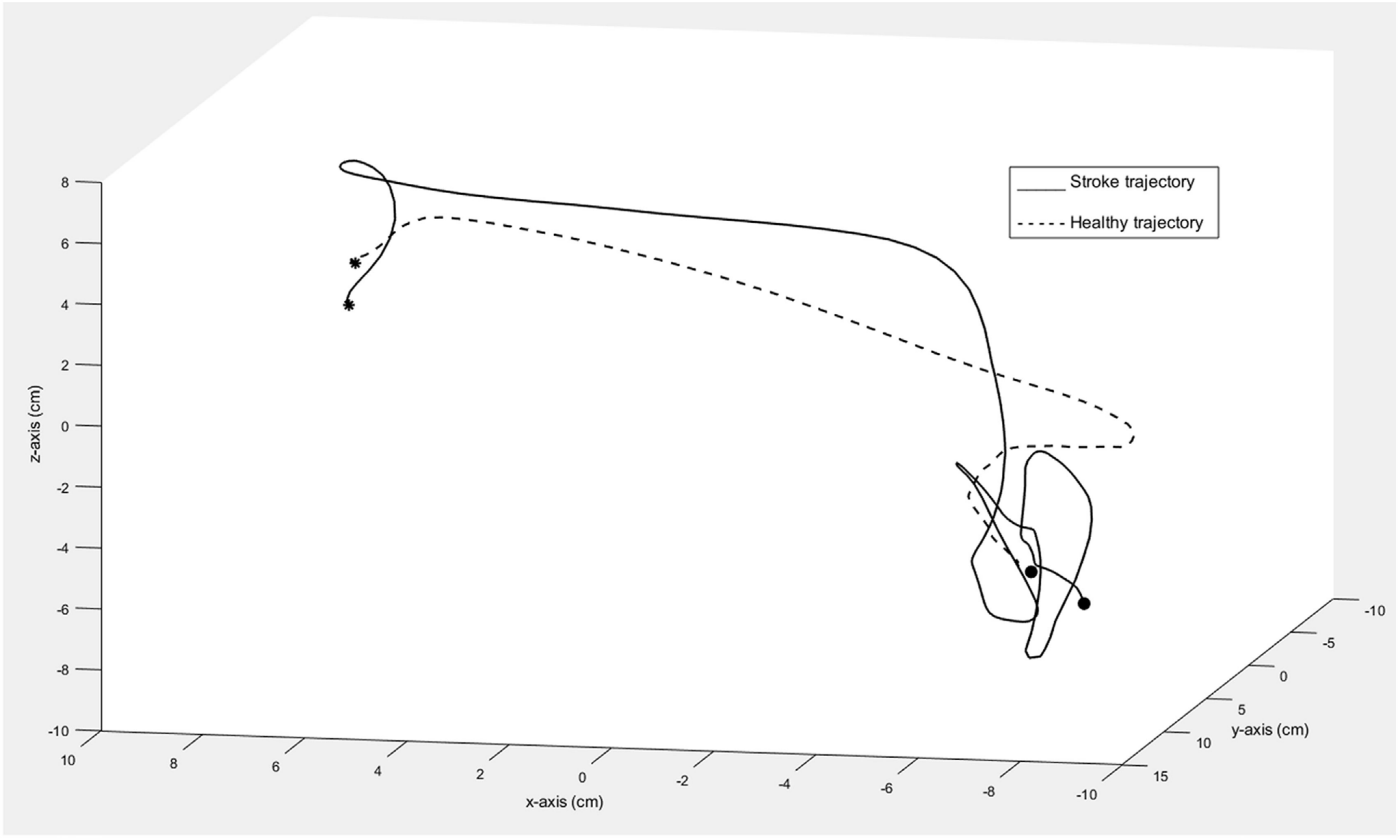
Trajectory of one movement segment in 3D space shown for one individual with moderate stroke impairment (solid line) and one healthy control (dashed line). The asterisk marks the start and the circle marks the end of the movement segment.

There was no significant difference between individuals with stroke and healthy controls in terms of relative time to peak velocity (*p* = 0.23, *r*_pb_ = 0.11). The graphical representation of movement data showed, however, that toward the end of the movement, the movement trajectory was frequently found to be clustered in individuals with stroke, resembling the appearance of a spider’s web (Figure 4). This altered movement strategy can also be observed on the velocity curves (Figure 3).

### Smoothness

There stroke group had significantly higher number of velocity peaks when compared to healthy controls (*p* < 0.001, *r*_pb_ = 0.57). The sensitivity was 64.2% and specificity was 83.7%. The differences in number of the velocity peaks were not significant between mild and moderate stroke groups (*p* = 0.03, *r*_pb_ = 0.26) but were significant between mild stroke group and healthy controls (*p* < 0.001, *r*_pb_ = 0.48) as well as moderate stroke group and healthy controls (*p* < 0.001, *r*_pb_ = 0.68).

## Discussion

This study shows that individuals with mild and moderate stroke use longer time to perform the target-to-target task with their stroke-affected arm in a VR environment. The movement time, mean and peak velocity measures all showed ability to discriminate between all groups except for peak velocity between mild stroke impairment and healthy controls. Individuals with stroke demonstrated a mean of 4.8 velocity peaks during each movement segment whereas healthy controls had a mean of 2.8 velocity peaks only. The number of velocity peaks effectively discriminated stroke subgroups from healthy controls. Thus, VR-enabled assessment is useful for discriminating between mild and moderate levels of upper limb impairments in stroke as well as between stroke impairment and healthy controls.

Longer movement times similar to the current study were found when individuals with chronic stroke performed reaching movements from a centrally located target to one of the eight peripheral targets located 10 cm away, using a robotic device, quickly and accurately (19). Although the effect sizes reported in the study were comparable to the results in the present study, they considered the ipsilesional limb as a reference in comparing between the arms. Longer movement times were also demonstrated for planar pointing movements of the hemiparetic arm in stroke (24) and in another smaller study using the VR enabled target-to-target pointing task (33). Movement time is frequently reported in fast pointing movements using motion capture systems and is considered as one of the key variables for kinematic movement analysis of upper limb tasks in stroke (17, 44, 45). In the present study, this finding is extended to target-to-target pointing task in a well-defined group of individuals with stroke. The large effect sizes found for movement time in the present study indicate that movement duration is one of the effective measures that discriminate between individuals with different levels of stroke impairment and healthy controls.

Mean velocity was found to discriminate between stroke impairment and healthy controls in an earlier study, where participants with stroke moved a cursor to the targets displayed on a screen using an exoskeleton that permits free arm movements (21). The finding from the present study that individuals with stroke have significantly lower peak velocity than healthy controls has been well described in literature (17, 19). On the other hand, the peak velocity of the affected arm was found to identify the least number of stroke participants (19–27%) as different from controls in a study where participants performed unassisted weight-supported horizontal reach-to-target movements using an exoskeleton as quickly and accurately as possible (22). In the present study, the lowest sensitivity of 46% to correctly discriminate between stroke group and controls was found for peak velocity. In this case, the sensitivity may have been influenced by the fact that the stroke group with mild impairment was not significantly different from controls. For moderate stroke alone, sensitivity was found to be 69.7% in the present study. These findings suggest that peak velocity might be sensitive to discriminate between moderate upper limb impairment and healthy controls rather than between mild impairment and controls.

Although individuals with stroke show slower movement times, the relative time to peak velocity did not differ significantly between the groups. The data from the present study indicate that individuals with stroke use similar movement strategy for this short target-to-target task even when their movements are slower and less smooth than healthy controls. The finding suggests that timing of the first fast visually triggered outward movement toward the target is relatively unaffected in individuals with stroke. In contrast, time to peak velocity was discriminative for stroke and healthy controls in a motion capture study involving self-paced, reach-to-grasp task in stroke (26). Even when the relative time to peak velocity did not show significant differences between groups, a different movement strategy was frequently observed among individuals with stroke. Here, the movement trajectory was found to be clustered toward the end of the movement, appearing like a spider’s web. These trajectory clusters reflect the difficulties in reaching the target in a single, continuous, and smooth movement, possibly due to the impaired motor control and feedback mechanism in stroke (33).

The number of velocity peaks was used as an indicator for smoothness of movement in this study. This is a count of sub-movements representing repetitive accelerations and decelerations used to complete the movement segment. This metric, also referred to as movement units, is frequently used in stroke kinematics, which has shown, similarly to our results, significant differences between upper limb movements in hemiparetic stroke and healthy subjects (21, 22, 26). As similar results have been obtained in studies using optoelectronic systems (17, 26), exoskeletons (19, 46), and now in VR-haptics, number of movement peaks proves to be independent of measurement device or task used and suitable for discrimination of impaired and non-impaired movement performance.

Kinematic trials have been recommended to be applied alongside clinical assessments to address questions about movement quality after stroke (6). In order to understand the meaning of changes in cortical activity following stroke, it is important to perform simultaneous, serial measurement of changes in motor performance as accurately as possible (47). In this context, kinematic discrimination between normal and more-affected stroke arm will be helpful in gaining more insights about motor patterns that are associated with cortical map re-organization.

The strength of this study is the use of a sensitive assessment method to differentiate among individuals with in mild and moderate stroke as well as healthy subjects. Another strength is the relatively large, unselected sample of this study and that 69% of participants were tested within 10 days of stroke onset. Thus, the results can be generalized to individuals in the acute and subacute stage after stroke. However, since the stroke group consisted only of individuals who were able to perform the target-to-target task, the findings from our study can be generalized only to those with mild to moderate stroke. Three participants with stroke reported as having used VR system a few times before their assessment session. This was taken to be too low for influencing the results from the VR assessment. As in all end-point kinematics measurements, the dynamic interaction between the components of the upper limb cannot be captured using this method. On the other hand, the target-to-target task is time-efficient and suitable for the clinical and community setting. It is relatively inexpensive, very easy to use, and requires lesser technical level of expertise when compared to other kinematic assessment methods such as optoelectronic camera systems and robotic exoskeletons. Similar to the experiences from the participants of this study, the target-to-target task has been previously described as enjoyable by the participants with stroke (33). The task’s ability to also discriminate between different levels of impairment is more informative while interpreting the results.

Hence, the VR-based target-to-target task appears to be a promising assessment tool for stroke. Further research needs to be done to evaluate the association of its outcome variables with clinical measures of upper limb impairment.

## Conclusion

The target-to-target task can provide valuable and specific information about sensorimotor impairment of the upper limb following stroke that might not be captured using traditional clinical scales. Therefore, this task can be useful for clinicians while planning the treatment in individuals with stroke.

## Availability of Data and Material

The datasets used and/or analyzed during the current study is not publicly available because further data analysis is ongoing. Requests to access the datasets should be directed to Netha Hussain (netha.hussain@gu.se).

## Ethics Statement

This study was carried out in accordance with the recommendations of Ethical Review Act, approved by The Regional Ethical Review Board in Gothenburg (No. 318-04, 225-08) with written informed consent from all subjects. All subjects gave written informed consent in accordance with the Declaration of Helsinki. The protocol was approved by the Regional Ethical Review Board in Gothenburg, Sweden.

## Author Contributions

All authors made substantial contributions to concept, design, acquisition, and interpretation of data. NH drafted the manuscript and MAM and KSS revised it critically for intellectual content. All authors read and approved the final manuscript.

## Conflict of Interest Statement

The authors declare that the research was conducted in the absence of any commercial or financial relationships that could be construed as a potential conflict of interest.

## Abbreviations

VR, virtual reality; SALGOT, stroke arm longitudinal study at Gothenburg University; FMA-UE, Fugl-Meyer assessment of upper extremity; ARAT, action research arm test; WHO, World Health Organization; IQR, interquartile range; 3D, three-dimensional.
